# Hyaluronic Acid/Platelet-Rich Plasma Mixture Improves Temporomandibular Joint Biomechanics: A Systematic Review

**DOI:** 10.3390/ijms25179401

**Published:** 2024-08-29

**Authors:** Maciej Chęciński, Karolina Lubecka, Filip Bliźniak, Dariusz Chlubek, Maciej Sikora

**Affiliations:** 1 Department of Oral Surgery, Preventive Medicine Center, Komorowskiego 12, 30-106 Kraków, Poland; maciej@checinscy.pl (M.C.); fblizniak@gmail.com (F.B.); 2 Department of Biochemistry and Medical Chemistry, Pomeranian Medical University, Powstańców Wielkopolskich 72, 70-111 Szczecin, Poland; sikora-maciej@wp.pl; 3 Department of Maxillofacial Surgery, Hospital of the Ministry of Interior, Wojska Polskiego 51, 25-375 Kielce, Poland

**Keywords:** hyaluronic acid, synovial fluid, temporomandibular joint, arthritis, temporomandibular disorders, platelet-rich plasma

## Abstract

Hyaluronic acid (HA) is the main component of the temporomandibular joint (TMJ) synovial fluid. Arthritis in temporomandibular disorders (TMDs) disrupts HA metabolism, resulting in shorter polymeric chain predominance and increased friction. Intra-articular injections of HA supplement the larger molecules of this glycosaminoglycan, and the platelet-rich plasma (PRP) delivered in this way releases growth factors, suppressing inflammation. This PRISMA-compliant PROSPERO-registered (CRD42024564382) systematic review aimed to assess the validity of mixing HA with PRP in the injectable treatment of TMJ disorders. We searched the medical literature for eligible randomized clinical trials using BASE, Google Scholar, PubMed and Scopus engines on 9 May 2024, with no time frame limit. Selected reports were assessed for risk of bias using the Cochrane RoB2 tool. Numerical data were collected on articular pain and mandibular mobility. We provided mean differences from baseline and between study and control groups at each observation point. The efficacy of TMD treatment with HA/PRP versus HA or PRP alone was assessed meta-analytically. Of 171 identified records, we selected 6 studies. In the 6-month follow-up, the mean advantage of PRP supplementation with HA was 2.52 (SE = 2.44; *d* = 0.83) mm and the benefit of adding PRP to HA was 1.47 (SE = 2.68; *d* = 0.34) mm in mandibular abduction. The pain-improvement scores were −1.33 (SE = 1.02; *d* = −1.05) and −1.18 (SE = 0.92; *d* = 0.80), respectively. Presumably, the HA/PRP range of therapeutic efficiency includes cases non-respondent to HA or PRP alone.

## 1. Introduction

**Figure 1:**
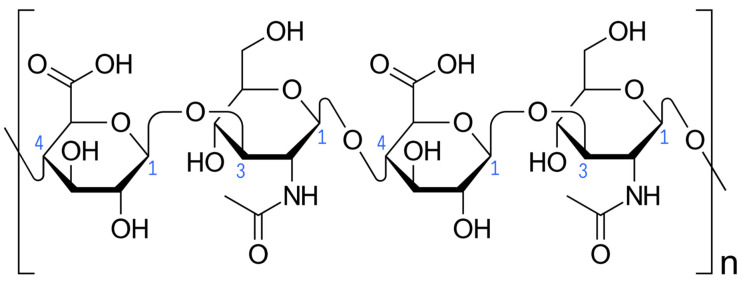
Hyaluronic acid (Haworth projection). Carbon atoms forming glycosidic bonds are numbered in blue. Square brackets and the letter “n” indicate multiplication of the illustrated fragment of the hyaluronic acid chain. Author: Vaccinationist. License: Public Domain.

Hyaluronic acid (HA) is the main component of the temporomandibular joint (TMJ) synovial fluid [1,2]. HA is synthesized in the synovial membrane by type B fibroblast-like synoviocytes from glucuronic acid and N-acetylglucosamine under hyaluronic acid synthasis (HAS) catalysis [3]. HASs convert simple sugars into the HA polymer linked via alternating β-(1→4) and β-(1→3) glycosidic bonds (Figure 1). This process occurs in the cell plasma membrane and HA is secreted into the extracellular space. By binding water, HA provides viscoelastic gel consistency to synovial fluid, reducing joint surface friction and dispersing loads [4]. Delivering newly synthesized HA to the synovial fluid improves the biomechanics of the TMJ. When HA degrades, the mechanical properties of the synovial fluid are weakened [5].

Mechanical overload, autoimmune diseases and infections lead to inflammation of the TMJ (arthritis), which disrupts HA metabolism [5]. The main mediators identified in arthritis are tumor necrosis factor-alpha (TNF-α), cytokines IL-1, IL-6 and IL-8 and matrix metalloproteinases (MMPs) [6,7,8]. The increase in the concentration of these factors stimulates HA synthesis in acute inflammation. A chronic one leads to damage of chondrocytes and subsequent degradation of cartilage tissue. This results in abundant and prolonged release of inflammatory mediators and induces inflammation of the adjacent parts of the synovial membrane (synovitis) [8,9]. Synovitis reduces HA production; hence, HA degradation predominates over its synthesis. Hyaluronidases and reactive oxygen species (ROSs) degrade HA polymer chains to oligosaccharides and low-molecular-weight HA [10,11]. A predominance of shorter-chained HA particles reduces TMJ lubrication, increasing friction and inflammation [2,9]. Clinically, this results in articular pain and TMJ hypomobility. Mobility limitation is observed in all directions [12]. It results from mechanical blockage, identified in the maximal mouth-opening test, and psychological blockage, expressed by painless mandibular abduction decrease [13].

Limited mandible abduction, protrusion and lateral movements imply a decline in mastication and overall health-related life quality [14,15,16]. Depending on the complexity of temporomandibular disorder (TMD) in a given case, a wide range of therapeutic methods are used. These fall into psychology, physiotherapy, pharmacotherapy, orthodontics, dental prosthetics and maxillofacial surgery [17,18]. The latter involves direct interference in the TMJ structures. Open surgery, endoscopic surgery (arthroscopy) and intra-articular injections are used, depending on the TMD type and severity [19,20,21]. Minimally invasive manipulations enable rinsing of the joint cavity and intra- and pericapsular administration of various substances.

Typically used injectables are dominated by (1) hyaluronic acid (HA) as a supplementing agent, (2) centrifuged blood products (CBPs), (3) drugs such as corticosteroids or non-steroidal anti-inflammatory drugs, (4) hypertonic dextrose and (5) unprocessed blood [22,23,24,25,26,27]. The latter two are irritants used to reduce the number of episodes of chronic jaw dislocation. Therefore, they have the opposite effect than the other substances mentioned [28,29]. All blood products are obtained as an autograft, typically from peripheral venous blood. Subsequent generations of CBPs differ in composition and are intended to better inhibit the progression of degeneration and even, to some extent, regenerate the cartilage of joint surfaces. These include but are not limited to plasma rich in growth factors (PRGF), platelet-rich plasma (PRP) and injectable platelet-rich fibrin (I-PRF) [30,31,32].

PRP is the most widely studied CBP TMJ injectable [23]. It is produced by centrifugation of blood, most often from the elbow bend, which allows the removal of the red blood cell fraction. PRP consists of plasma and platelets. Plasma contains water, electrolytes, proteins, hormones and enzymes. Platelets are the source of growth factors with anti-inflammatory and regenerative effects. This group includes (1) platelet-derived growth factor (PDGF), (2) transforming growth factor-beta (TGF-β), (3) vascular endothelial growth factor (VEGF), (4) epidermal growth factor (EGF), (5) fibroblast growth factor (FGF) and (6) insulin-like growth factor (IGF) [33,34].

HA is commercially available, which means it is practically unlimited. It can be stored for a long time, according to the manufacturer’s recommendations. In turn, the availability of PRP varies depending on the quality of the patient’s blood, the experience of the staff and the correctness of the centrifugation procedure. There is a risk of complete failure in the attempt to obtain PRP. In addition, treatment with PRP requires an additional blood-collection procedure, and the product must be used immediately. In the case of a series of intra-articular injections, blood must be collected and centrifuged at each visit. These inconveniences do not apply to HA.

Knowledge regarding minimally invasive manipulations inside the TMJs is changing [35]. The search for the most regeneration-promising injectable has been directed towards autografts [29,36]. Concerning TMJ lubrication, medium- and high-molecular-weight HA supplementation remains irreplaceable [37,38]. A current review aimed at searching for new solutions in the injection treatment of TMJs showed the existence of clinical studies presenting the intra-articular administration of a mixture of CBPs and HA [35]. This means that the theoretically reasonable combination of the benefits of both substances is beginning to be clinically tested. The results of these tests could be interesting and, in the best-case scenario, revolutionize the choice of injectable substances. The low evidence of a single clinical trial, even among the best-designed trials, encourages the synthesis and meta-analysis of the results of all available research on the topic.

This systematic review aimed to assess the effectiveness of administering CBPs and HA mixtures to the TMJ cavity regarding mandibular mobility range, articular pain and health-related quality of life. Intra-articular administrations of placebo or any other injectable were assessment reference points.

## 2. Results

### 2.1. Selection

**Figure 2:**
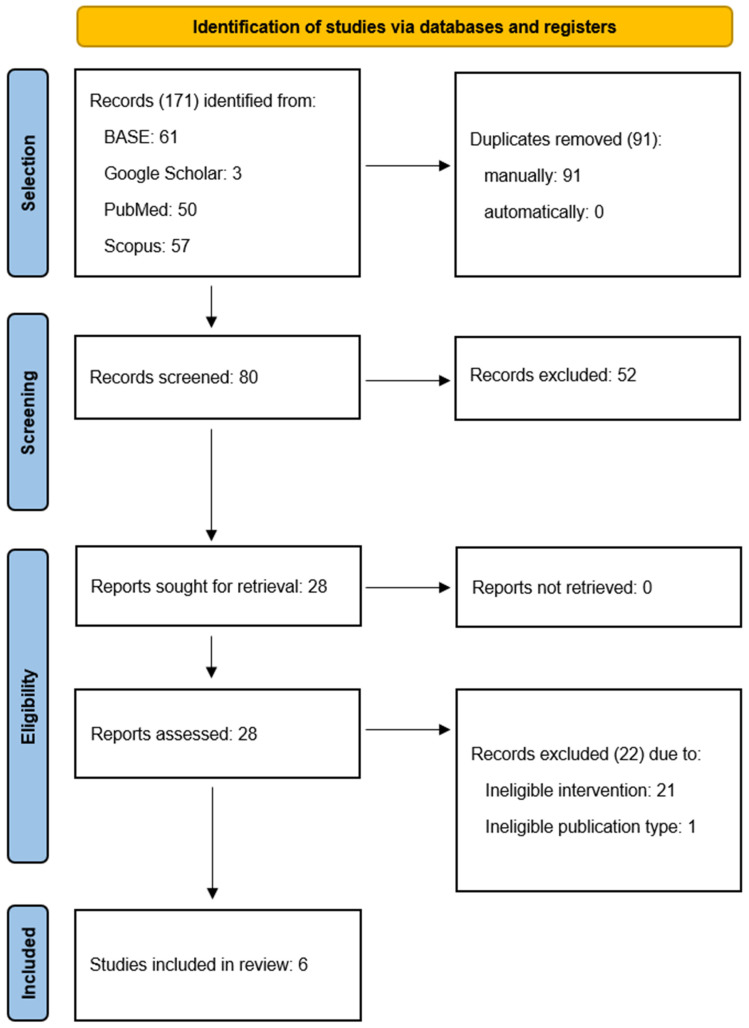
Flow diagram.

Of the 171 records identified, 91 were excluded due to duplication and 80 were screened (Figure 2). Of the 28 that underwent full-text evaluation, 22 were excluded with reasons provided (Table A1) and 6 were included in the review (Table 1). The inter-rater agreement was 93.75% and κ = 0.86.

The included studies are characterized in Table 2. The diagnosis was limited to TMD or osteoarthritis or specified the type of internal derangement. The number of subjects ranged from 10 to 30 per group. Most research teams opted for arthrocentesis before injection. Controls included HA alone, PRP alone or corticosteroids. Follow-up was at least 6 months, allowing for meta-analysis of 6-month variable values.

**Figure 3:**
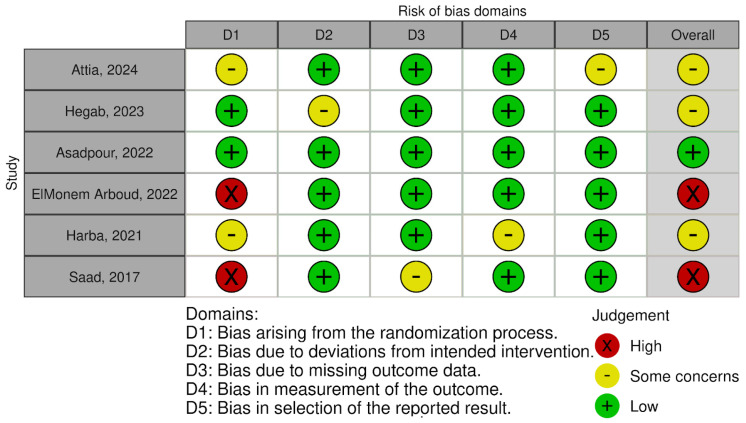
Risk of bias–traffic light plot [39,40,41,42,43,44].

**Figure 4:**
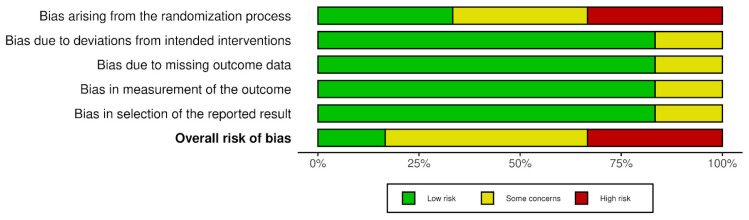
Risk of bias–summary plot.

The risk of bias ranged from low, through some concerns, to high, as detailed in Figure 3 and Figure 4.

### 2.2. Individual Studies

Mandibular abduction was assessed in all eligible studies (Table 3). Mean baseline values were collected for all patient groups. The longest available follow-up period was 12 months. The most numerous mandibular abduction variables were extracted for the 1- and 6-month follow-ups. During the first month, some investigators conducted more frequent check-ups. Apart from one study comparing the HA plus PRP group with the PRP alone group, no statistically significant differences were observed in the initial values. They ranged from an average of 23 to an average of 36.5 mm. The smallest mean mouth opening at the end of treatment in the group of patients was 31 mm in patients treated for TMJ osteoarthritis in the trial of Hegab et al. [40]. The treatment in this group was performed by combining arthrocentesis with intra-articular HA injection and achieved an average of 8 mm of improvement [40].

Apart from one receiving HA alone in the study by Harba et al., all patient groups achieved a statistically significant mean improvement in mouth-opening range [43]. After 6 months, both available studies noted statistically significant differences in the treatment results in favor of the HA plus PRP mixture versus PRP alone. Assessing the change in mandibular abduction gave contradictory results when comparing HA with PRP versus HA alone. One study showed statistical significance of such a difference in favor of the HA + PRP mixture, the remaining three presented no statistical significance of the differences between the means for the groups treated with HA + PRP and those receiving HA.

The availability of pretreatment and follow-up values for the articular pain variable was identical to that for the mandibular abduction (Table 4). In each study, the differences in the initial values between the groups were not statistically significant. Mean pain started at a minimum of 6.5 points and decreased to a maximum of 3.5 points. In the groups treated with the HA and PRP mixture, the highest mean pain score at the end of the follow-up was 2.25 points.

Each treatment provided a statistically significant improvement compared to the pre-intervention assessment. After 6 months, the differences between HA and PRP treatment and PRP alone were statistically significant in both available studies. Two trials proved, and two others did not present, statistically significant differences between treatment with both substances and HA alone.

None of the eligible clinical trials assessed health-related quality of life.

### 2.3. Syntheses

The range of mandibular mobility expressed as abduction values decreased significantly in each group receiving the HA/PRP mixture. The initial intergroup mean values of mouth opening were 29.14 (SD = 5.26), 27.40 (SD = 5.28) and 25.20 (SD = 2.69) mm for the HA/PRP, HA alone and PRP alone groups, respectively. At 1-month follow-up, the mean values were 37.90 (SD = 4.97), 36.68 (SD = 4.11) and 33.45 (SD = 1.20) mm for those treated with HA plus PRP, HA and PRP, respectively. At 6 months, abduction values were 41.51 (SD = 6.61), 38.30 (SD = 5.05) and 35.05 (SD = 5.02) mm according to the same order of injectables.

**Figure 5:**
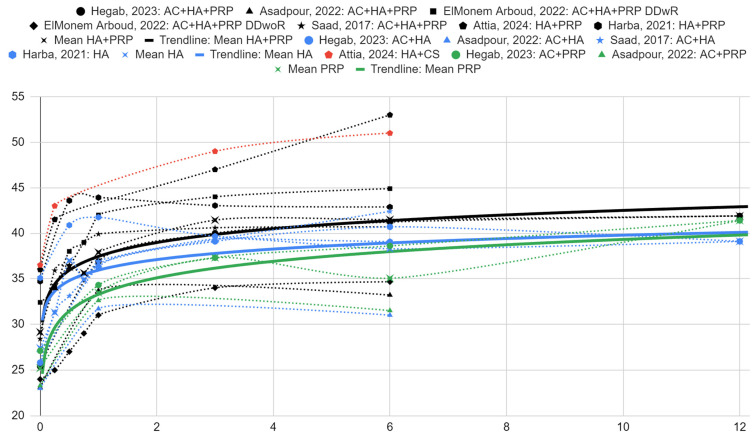
Mandibular abduction in millimeters over time (months) [39,40,41,42,43,44].

Figure 5 illustrates the in-question variable values for individual patient groups and relative time points. As in the case of the articular pain variable, trend lines were drawn for the mean values for the groups treated with individual agents. Good fits of the logarithmic regression model were obtained in each case, i.e., for HA with PRP (R^2^ = 0.91), HA only (R^2^ = 0.69), and PRP only (R^2^ = 0.65).

The difference in the mean intergroup treatment results between HA with PRP and HA after 1 month was 1.22 (SE = 3.01) mm. The analogous mean difference compared to patients treated with PRP alone was 4.45 (SE = 3.73) mm. In both comparisons, the differences favored the therapy with the mixture of both substances. However, they were not statistically significant. After 6 months, the difference in HA with PRP treatment was observed to be 3.21 (SE = 3.85) mm compared to HA alone and 6.46 (SE = 5.14) mm compared to PRP injection alone. These were also not statistically significant.

In each group of patients, there was a statistically significant decrease in the intensity of articular pain on a 0–10 scale. Initially, the mean intergroup values were 7.14 (SD = 0.51), 6.93 (SD = 0.31) and 7.20 (SD = 0.28) in the patient group treated with HA and PRP, HA alone and PRP alone, respectively. After 1 month, the means were 1.77 (SD = 0.73), 2.32 (SD = 1.12) and 4.65 (SD = 3.46), and after 6 months, 0.90 (SD = 0.89), 1.87 (SD = 1.61) and 2.30 (SD = 0.99) for the HA and PRP, HA alone and PRP alone groups, respectively.

**Figure 6:**
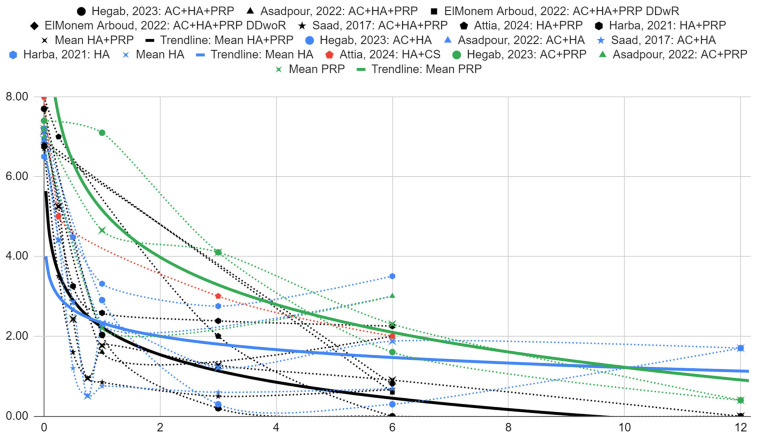
Articular pain on a 0–10 scale over time (months) [39,40,41,42,43,44].

The graphs of articular pain intensity scores before treatment and at follow-up for the synthesized HA with PRP, HA and PRP groups are illustrated in Figure 6. The proposed logarithmic regression model fits the data well for PRP (*R*^2^ = 0.89) and HA with PRP (*R*^2^ = 0.66). Despite the poor fit, a trend line for the HA groups (*R*^2^ = 0.29) is presented for comparison.

There is an observed difference in intergroup means between articular pain scores for the HA with PRP, HA and PRP groups. They are −0.55 (SE = 0.67) and −2.88 (SE = 1.60) in favor of HA with PRP over HA and PRP, respectively, at 1 month of follow-up, but are not statistically significant. At 6 months, they are −0.97 (SE = 1.01) and −1.40 (SE = 1.23) for HA and PRP over HA with PRP, respectively, albeit also not statistically significant.

Summarizing the results, Table 5 presents the discrepancies in mandibular abduction increase and articular pain relief between HA/PRP treatment versus HA or PRP control in 6-month observation. All comparisons include randomized controlled trials of various risks of bias.

## 3. Discussion

### 3.1. Interpretation

Treatment with HA, PRP or their combination resulted in a mandibular abduction increase and articular pain intensity decrease in the study as well as control groups [39,40,41,42,43,44]. This means that, compared to preintervention ones, the mean scores improved in each patient group, but did not necessarily in every patient. TMDs constitute a large group of diseases, which, despite similar symptoms and a similar clinical course, differ in etiology and may require different therapeutic approaches [20,45,46].

Currently, arthritis, disc displacement, degenerative joint disease and subluxation are distinguished as diagnoses manifesting articular pain [20,45,46]. Arthritis is a broad category of manifestations of specific diseases, which always requires a search for the cause of inflammation. Identification of the cause allows for the proper direction of treatment towards its removal. Among internal derangement diagnoses, the displacement of the disc with and without reduction predominates. It has been proven that intra-articular injections bring relief in both cases, but despite this, these are different clinical situations that usually qualify for combined treatment [42]. Degenerative disease requires identification and removal or limitation of the cause. The possibility of its reversal at the tissue level is also seen in autografts of mesenchymal stem cells. In habitual dislocation, intra-articular injections limit the number of dislocation episodes. In such cases, irritating preparations are used [28,29].

Among the complex therapeutic strategies, injection therapy is only one of many elements [17,18,19,47,48]. It is particularly important in cases presenting TMJ hypomobility and arthritis [26]. The first clinical presentation can be solved by HA supplementing, especially medium- or high-molecular-weight [2,38,49]. The second diagnosis requires ad hoc suppression of inflammation, which can be achieved by systemic pharmacotherapy or as an intra-articular PRP mechanism of action element [13,50]. In the long term, inflammation-recurrence reduction is achieved by removing the causative factor and regenerating articular cartilage [7,31,48,51,52,53]. Growth factors in CBPs support the regenerative process [33,34].

The diagnosis of TMD often combines mandibular hypomobility with arthritis as a causative factor [18,26]. However, this does not exclude the coexistence of disc displacement or degenerative joint disease [20,45,46]. The results of this systematic review prove that the HA/PRP mixture improves TMJ movements and resolves articular pain better than HA or PRP alone in almost every patient group tested. Presumably combined therapy effectiveness spectrum covers cases non-respondent to HA or PRP alone.

### 3.2. Limitations

The source studies were not homogeneous in terms of diagnosis. Intra-articular administration of hyaluronic acid has an important lubricating effect, and injection of autologous blood products has regenerative potential. While in most cases of TMDs, both mechanisms are desirable, the severity of hypomobility and degeneration are elements that make up the diagnosis [45,46,54,55,56,57]. It would be more appropriate to conduct separate meta-analyses, for disk displacement with or without reduction, arthritis and degenerative joint disease [56,57,58]. However, in this systematic review, the limited number of studies precludes dividing them into subgroups.

The search was conducted in English, which may have resulted in missing records unindexed with English keywords. Due to the scarcity of the source material, studies with uncertain and with high risk of bias were included in the synthesis.

### 3.3. Strengths

The strengths of this systematic review include (1) detailed eligibility criteria, (2) comprehensiveness of the search, (3) adherence to PRISMA guidelines and (4) meta-analytic evaluation of treatment effectiveness in both articular pain and mandibular mobility domains.

### 3.4. Implications

HA with PRP presents better mandibular mobility improvement than using either of these substances separately. However, these are differences based on small numbers of study groups and between means with high standard deviations. Testing the statistical significance of these differences gave a negative result (*p* > 0.05). Also, the mean pain relief efficacy discrepancy in the groups of patients treated with HA and PRP is not statistically significant compared to the HA or PRP control (*p* > 0.05).

Nevertheless, the differences in the best documented 1- and 6-month follow-up periods are noticeable and in favor of the mixture of both injectables. Hence, the lack of statistical significance was probably also due to the small number of patient groups and large discrepancies between them. This justifies further research into combining HA and centrifuged blood products in TMD treatment.

## 4. Materials and Methods

### 4.1. Protocol and Criteria

This systematic review with meta-analysis was conducted following the Preferred Reporting Items for Systematic Reviews and Meta-Analyses PRISMA 2020 and registered in the PROSPERO International Prospective Register of Systematic Reviews database under number CRD42024564382 [59].

The eligibility criteria were developed using the SPIDER tool (version 2012.07.24; Central Manchester NHS Foundation Trust, Manchester, UK) and are presented in Table 6 [60].

### 4.2. Searches

Final searches in medical research article databases were performed on May 9, 2024, using the following engines: (1) Bielefeld Academic Search Engine (BASE; Bielefeld University Library, Bielefeld, Germany); (2) Google Scholar (Google LLC, Mountain View, CA, USA); (3) PubMed (National Library of Medicine, Bethesda, MD, USA); (4) Scopus (Elsevier, Amsterdam, Netherlands). There were no limits on the time frame for searches.

The search strategy was based on preliminary searches and the current mapping review [23]. The query “temporomandibular AND (hyaluronic OR hyaluronan OR ha) AND (blood OR plasma OR fibrin OR prgf OR prp OR prf OR i-prf OR lpcgf) AND (intra-articular OR intra articular OR injection)” was applied to all search engines. In the case of the Google Scholar engine, the search was performed on title content only, as the unfiltered results provided by this search engine go beyond the scope of the query.

### 4.3. Selection and Collection

Identified records from all engines were input to the Rayyan automation tool (version 2024-08-08, Qatar Computing Research Institute, Doha, Qatar and Rayyan Systems, Cambridge, MA, USA) [61]. Then the manual deduplication was performed by M.C. Records that successfully underwent the stages above and were subsequently assessed blindly in terms of suitable titles and abstracts by two independent researchers (K.L. and F.B.). Cohen’s kappa agreement between researchers was calculated with the MedCalc Inter-rater agreement tool (version 22.032, MedCalc Software Ltd., Ostend, Belgium). Records were promoted to the next stage of full-text evaluation in case of assessors’ disagreement.

Two independent researchers (M.C. and K.L.) performed the data collection stage. On inconsistency, a consensus was made. If necessary, a casting voice of a third researcher (M.S.) was asked. For this process, no automation tool was used. The tables presenting and comparing numerical data were prepared using the Google Workspace package (version 2024.07.06, Google LLC, Mountain View, CA, USA).

Characterizing data included (1) diagnosis, (2) study group number of patients, (3) study group number of TMJs, (4) study group substance, (5) control group number of patients, (6) control group number of TMJs, (7) control group substance and (8) follow-up duration. To determine the effectiveness of the therapy, numerical data regarding (1) the extent of mandibular abduction, (2) articular pain and (3) health-related quality of life were extracted. Imaging or navigation were not considered, despite their supposed benefits in degenerative joint disease diagnosis and invasive TMD treatment [62].

Pain was transformed to a scale of 0–10, regardless of how its intensity was assessed. Mandibular mobility was determined as a value in millimeters. Depending on availability, the items collected were maximum unassisted abduction, maximum manually assisted abduction and maximum pain-free abduction. If the previously mentioned variable was unavailable, the next one was included. Regardless of the measurement method, the life-quality score was intended to be converted into a percentage. Variable values were extracted for baseline ones and all available follow-up periods.

### 4.4. Assessments

The risk of bias was determined using the Cochrane RoB 2: A revised Cochrane risk-of-bias tool for randomized trials (version 2019.08.22, The Cochrane Collaboration, London, UK) [63]. The results of this evaluation were visualized using the Robvis tool (version 2023, University of Bristol, Bristol, UK) [64,65].

The effect measures included mean differences between the values during observation and the baseline and between the values for the study and control groups at a given observation point. These were calculated with MedCalc software (version 22.023, MedCalc Software Ltd., Ostend, Belgium) and the Practical Meta-Analysis Effect Size Calculator tool (version 2023.11.27, George Mason University, Philadelphia, PA, USA).

Only randomized controlled trials were included in the synthesis. Raw data and mean differences were tabulated and presented in graphs. Google Workspace software (version 2024-06-28, Google LLC, Mountain View, CA, USA) was used for visualization.

Certainty was assessed by calculating the effect size (Cohen’s *d*) and determining the characteristics of patient groups for individual effect measures with the Practical Meta-Analysis Effect Size Calculator tool (version 2023.11.27, George Mason University, Philadelphia, PA, USA).

## 5. Conclusions

The only CBP currently combined with HA in the TMJ injection treatment is PRP. HA/PRP treatment results are beneficial compared to the initial mandibular abduction and articular pain values. The improvements are quantitatively superior to administering HA or PRP alone, yet statistically insignificant most likely due to the small number of patient groups analyzed. Presumably, the HA/PRP range of therapeutic efficiency includes cases non-respondent to HA or PRP alone.

## Figures and Tables

**Table 1:** Included reports.

First Author	Publication Year	Digital Object Identifier or PubMed Identifier	Title
Attia [39]	2024	https://doi.org/10.1007/s12663-023-01907-6	Hyaluronic Acid and Platelet-Rich Plasma Mixture Versus Hyaluronic Acid and Corticosteroid in the Treatment of Temporomandibular Joint Internal Derangement: A Comparative Randomized Study
Hegab [40]	2023	https://doi.org/10.1016/j.jormas.2022.11.016	Synergistic effect of platelet rich plasma with hyaluronic acid injection following arthrocentesis to reduce pain and improve function in TMJ osteoarthritis
Asadpour [41]	2022	https://doi.org/10.1016/j.joms.2022.05.002	Combined Platelet-Rich Plasma and Hyaluronic Acid can Reduce Pain in Patients Undergoing Arthrocentesis for Temporomandibular Joint Osteoarthritis
ElMonem Arboud [42]	2022	https://doi.org/10.4103/tdj.tdj_5_22	Evaluation of intraarticular injection of hyaluronic acid with platelet rich plasma for treatment of temporomandibular joint anterior disc displacement with and without reduction
Harba [43]	2021	https://doi.org/10.17219/dmp/127446	Evaluation of the participation of hyaluronic acid with platelet-rich plasma in the treatment of temporomandibular joint disorders
Saad [44]	2017	https://doi.org/10.21608/edj.2017.76108	Intra articular injection of hyaluronic acid alone in comparison with hyaluronic acid and PRP in the treatment of internal derangement of temporomandibular joint

**Table 2:** Study characteristics.

First Author, Publication Year	Diagnosis	Study Group Number of Patients	Study Group Substance	Control Group Number of Patients	Control Group Substance	Follow-Up Duration
Attia, 2024 [39]	Disk displacement with reduction	30	HA + PRP	30	HA + CS	6 months
Hegab, 2023 [40]	TMJ osteoarthritis	30	AC + HA + PRP	30	AC + HA	12 months
				30	AC + PRP	
Asadpour, 2022 [41]	TMJ osteoarthritis	10	AC + HA + PRP	10	AC + HA	6 months
				10	AC + PRP	6 months
ElMonem Arboud, 2022 [42]	Disk displacement with reduction	10	AC + HA + PRP	0	N/A	6 months
	Disk displacement without reduction	10	AC + HA + PRP	0	N/A	6 months
Harba, 2021 [43]	Temporomandibular disorders	12	HA + PRP	12	HA	6 months
Saad, 2017 [44]	Disk displacement	10	AC + HA + PRP	10	AC + HA	6 months

AC—arthrocentesis; CS—corticosteroid; HA—hyaluronic acid; N/A—not applicable; N/S—not specified; PRP—platelet-rich plasma; TMJ—temporomandibular joint.

**Table 3:** Mandibular abduction in millimeters.

First Author, Publication Year	Group, Size	Baseline	1 Week	2 Weeks	3 Weeks	1 Month	3 Months	6 Months	12 Months
Attia, 2024 [39]	HA + PRP, 30	36.00	41.50	N/S	N/S	N/S	47.00	53.00 *	N/S
	HA + CS, 30	36.50	43.00	N/S	N/S	N/S	49.00	51.00 *	N/S
	Mean difference	−0.50 †	−1.50	N/S	N/S	N/S	−2.00	2.00 †	N/S
Hegab, 2023 [40]	AC + HA + PRP, 30	25.40 SD = 3.10	N/S	N/S	N/S	36.90 SD = 3.20	40.00 SD = 2.70	41.23 * SD = 2.50	41.90 SD = 2.20
	AC + HA, 30	25.80 SD = 3.20	N/S	N/S	N/S	36.30 SD = 2.20	39.10 SD = 1.80	40.70 * SD = 2.06	39.10 SD = 2.80
	Mean difference	−0.40 † SE = 0.81	N/S	N/S	N/S	0.60 † SE = 0.71	0.90 † SE = 0.59	0.53 † SE = 0.59	2.80 * SE = 0.65
	AC + PRP, 30	27.10 SD = 2.90	N/S	N/S	N/S	34.30 SD = 2.80	37.30 SD = 2.30	38.60 * SD = 1.50	41.40 SD = 2.70
	Mean difference	−1.70 * SE = 0.78	N/S	N/S	N/S	2.60 * SE = 0.78	2.70 * SE = 0.65	2.63 * SE = 0.53	0.50 † SE = 0.64
Asadpour, 2022 [41]	AC + HA + PRP, 10	23.10 SD = 2.40	N/S	N/S	N/S	33.70 SD = 1.70	N/S	33.20 * SD = 1.60	N/S
	AC + HA, 10	23.00 SD = 2.30	N/S	N/S	N/S	31.70 SD = 1.60	N/S	31.00 * SD = 1.60	N/S
	Mean difference	0.10 † SE = 1.05	N/S	N/S	N/S	2.00 * SE = 0.74	N/S	2.20 * SE = 0.72	N/S
	AC + PRP, 10	23.30 SD = 2.50	N/S	N/S	N/S	32.60 SD = 1.70	N/S	31.50 * SD = 1.50	N/S
	Mean difference	−0.20 † SE = 1.10	N/S	N/S	N/S	1.10 † SE = 0.76	N/S	1.70 * SE = 0.69	N/S
ElMonem Arboud, 2022 [42]	AC + HA + PRP DDwR, 10	32.40 SD = 1.96	34.00	38.00	39.00	42.00	44.00	44.90 * SD = 1.20	N/S
	AC + HA + PRP DDwoR, 10	24.00 SD = 6.50	25.00	27.00	29.00	31.00	34.00	34.66 * SD = 10.59	N/S
Harba, 2021 [43]	HA + PRP, 12	34.71 SD = 9.42	N/S	43.58 SD = 5.52	N/S	43.92 SD = 6.78	43.04 SD = 7.26	42.88 * SD = 7.16	N/S
	HA, 12	35.08 SD = 10.54	N/S	40.88 SD = 7.14	N/S	41.75 SD = 6.09	39.67 SD = 5.05	39.08 † SD = 6.31	N/S
	Mean difference	−0.37 †	N/S	2.70 † SE = 2.61	N/S	2.17 † SE = 2.63	3.37 † SE = 2.55	3.80 † SE = 2.76	N/S
Saad, 2017 [44]	AC + HA + PRP, 10	28.40 SD = 2.07	35.90 SD = 2.42	36.50 SD = 1.65	38.90 SD = 1.52	39.90 SD = 1.52	40.60 SD = 1.52	40.70 * SD = 1.42 ‡	N/S
	AC + HA, 10	25.70 SD = 3.50	31.30 SD = 2.75	33.10 SD = 2.28	34.90 SD = 1.52	36.95 SD = 1.42	39.30 SD = 3.54	42.40 * SD = 3.44	N/S
	Mean difference	2.70 † SE = 1.29	4.60 * SE = 1.16	3.40 * SE = 0.89	4.00 * SE = 1.68	2.95 * SE = 0.66	1.30 † SE = 1.22	−1.70 † SD = 1.18	N/S

AC—arthrocentesis; CS—corticosteroid; HA—hyaluronic acid; PRP—platelet-rich plasma; DD—disk displacement; DDwR—disk displacement with reduction; DDwoR—disk displacement without reduction; SD—standard deviation; SE—standard error; *—statistically significant (*p* < 0.05); †—no statistical significance (*p* ≥ 0.05); ‡—source data inconsistency. The statistical significance indicator given with the values after 6 months refers to the difference between them and the baselines.

**Table 4:** Articular pain on a 0–10 scale.

First Author, Publication Year	Group, Size	Baseline	1 Week	2 Weeks	3 Weeks	1 Month	3 Months	6 Months	12 Months
Attia, 2024 [39]	HA + PRP, 30	8.00	7.00	N/S	N/S	N/S	2.00	0.00 *	N/S
	HA + CS, 30	8.00	5.00	N/S	N/S	N/S	3.00	2.00 *	N/S
	Mean difference	0.00 †	2.00	N/S	N/S	N/S	−1.00	−2.00 *	N/S
Hegab, 2023 [40]	AC + HA + PRP, 30	7.70 SD = 0.90	N/S	N/S	N/S	2.03 SD = 0.80	0.20 SD = 0.40	0.00 * SD = 0.00	0.00 SD = 0.00
	AC + HA, 30	7.20 SD = 1.40	N/S	N/S	N/S	2.90 SD = 0.90	0.30 SD = 0.50	0.30 * SD = 0.55	1.70 SD = 1.40
	Mean difference	0.50 † SE = 0.30	N/S	N/S	N/S	−0.87 * SE = 0.22	−0.10 † SE = 0.12	−0.30 * SE = 0.10	−1.70 * SE = 0.26
	AC + PRP, 30	7.40 SD = 1.10	N/S	N/S	N/S	7.10 SD = 1.20	4.10 SD = 1.06	1.60 * SD = 0.80	0.40 SD = 0.70
	Mean difference	0.30 † SE = 0.26	N/S	N/S	N/S	−5.07 * SD = 0.26	−3.90 * SD = 0.21	−1.60 * SE = 0.15	−0.40 * SE = 0.13
Asadpour, 2022 [41]	AC + HA + PRP, 10	7.10 SD = 0.50	N/S	N/S	N/S	1.60 SD = 0.50	N/S	2.00 * SD = 0.80	N/S
	AC + HA, 10	7.10 SD = 0.80	N/S	N/S	N/S	2.30 SD = 0.70	N/S	3.00 * SD = 0.80	N/S
	Mean difference	0.00 † SE = 0.30	N/S	N/S	N/S	−0.70 * SE = 0.27	N/S	−1.00 * SE = 0.36	N/S
	AC + PRP, 10	7.00 SD = 0.70	N/S	N/S	N/S	2.20 SD = 0.60	N/S	3.00 * SD = 1.20	N/S
	Mean difference	0.10 † SE = 0.27	N/S	N/S	N/S	−0.60 * SE = 0.25	N/S	−1.00 * SE = 0.46	N/S
ElMonem Arboud, 2022 [42]	AC + HA + PRP DDwR, 10	6.90 SD = 0.99	N/S	N/S	N/S	N/S	N/S	0.60 * SD = 0.52	N/S
	AC + HA + PRP DDwoR, 10	6.80 SD = 1.04	N/S	N/S	N/S	N/S	N/S	0.80 * SD = 0.79	N/S
Harba, 2021 [43]	HA + PRP, 12	6.75 SD = 2.46	N/S	3.25 SD = 1.02	N/S	2.58 SD = 1.88	2.38 SD = 2.09	2.25 * SD = 2.11	N/S
	HA, 12	6.50 SD = 1.65	N/S	4.48 SD = 1.57	N/S	3.31 SD = 1.72	2.75 SD = 1.22	3.50 * SD = 1.35	N/S
	Mean difference	2.25 † SE = 0.86	N/S	−1.23 * SD = 0.54	N/S	−0.73 † SD = 0.74	−0.37 † SD = 0.70	−1.25 † SE = 0.72	N/S
Saad, 2017 [44]	AC + HA + PRP, 10	6.70 SD = 1.34	3.50 SD = 0.71	1.60 SD = 0.70	0.95 SD = 0.44	0.85 SD = 0.47	0.50 SD = 0.53	0.67 * SD = 0.53	N/S
	AC + HA, 10	6.90 SD = 1.20	4.40 SD = 0.97	1.20 SD = 0.79	0.51 SD = 0.71	0.75 SD = 0.69	0.60 SD = 0.43	0.69 * SD = 0.51	N/S
	Mean difference	−0.20 † SE = 0.57	−0.90 * SD = 0.38	0.40 † SD = 0.33	0.44 † SD = 0.26	0.10 † SD = 0.26	−0.10 † SD = 0.22	−0.02 † SE = 0.23	N/S

AC—arthrocentesis; CS—corticosteroid; HA—hyaluronic acid; PRP—platelet-rich plasma; DD—disk displacement; DDwR—disk displacement with reduction; DDwoR—disk displacement without reduction; SD—standard deviation; SE—standard error; *—statistically significant (*p* < 0.05); †—no statistical significance (*p* ≥ 0.05). The statistical significance indicator given with the values after 6 months refers to the difference between them and the baselines.

**Table 5:** Summary of findings at 6-month follow-up.

Comparison	Number of Studies and Study/Control Groups	Total Number of Subjects in Study/Control Groups	Mean Difference with Standard Error, Statistical Significance and Confidence Interval	Effect Size with Standard Error and 95% Confidence Interval
Mandibular abduction in HA + PRP versus HA treatment	6 7/4	112/62	1.47 SE = 2.68; *p* = 0.60 −4.59–7.53	0.34 SE = 0.63 −0.89–1.58
Mandibular abduction in HA + PRP versus PRP treatment	6 7/2	112/40	2.52 SE = 2.44; *p* = 0.34 −3.24–8.28	0.83 SE = 0.83 −0.79–2.45
Articular pain in HA + PRP versus HA treatment	6 7/4	112/62	−1.18 SE = 0.92; *p* = 0.23 −3.27–0.91	−0.80 SE = 0.65 −2.07–0.47
Articular pain in HA + PRP versus PRP treatment	6 7/2	112/40	−1.33 SE = 1.02; *p* = 0.23 −3.74–1.08	−1.05 SE = 0.84 −2.69–0.60

**Table 6:** Eligibility criteria.

	Criteria for Inclusion	Criteria for Exclusion
Sample	Patients presenting temporomandibular joint disorders	Non-human research, cadaver research
Phenomenon of interest	Injections of HA mixed with blood product into the temporomandibular joints	Extracapsular injections
Design	Randomized clinical trials (applicable to meta-analysis)	Not applicable
Evaluation	Range of mandibular abduction, temporomandibular joint pain, health-related quality of life	Descriptive results only
Research type	Quantitative applied research	Not applicable

## Data Availability

Data is contained within the article.

## Appendix A

**Table A1:** Excluded reports.

First Author	Publication Year	Digital Object Identifier or PubMed Identifier	Title	Reasons for Exclusion
Yuan [66]	2024	https://doi.org/10.6084/m9.figshare.25324966.v1	Comparison of Temporomandibular Joint Osteoarthritis Treatment Efficacy of Platelet-rich Plasma and Hyaluronic Acid Injection in Upper or Lower TMJ Space	Ineligible intervention
Liu [67]	2023	https://doi.org/10.1016/j.jcms.2023.09.014	Platelet-rich plasma therapy for temporomandibular joint osteoarthritis: A randomized controlled trial	Ineligible intervention
Shan [68]	2023	https://doi.org/10.57760/sciencedb.o00013.00022	Platelet-rich plasma and hyaluronic acid for Injection treatment of temporomandibular joint degeneration	Ineligible intervention
Vingender [69]	2023	https://doi.org/10.1016/j.jcms.2023.01.017	Evaluation of the efficiency of hyaluronic acid, PRP and I-PRF intra-articular injections in the treatment of internal derangement of the temporomandibular joint: A prospective study	Ineligible intervention
Dasukil [70]	2022	https://doi.org/10.1016/j.jcms.2022.10.002	Intra-articular injection of hyaluronic acid versus platelet-rich plasma following single puncture arthrocentesis for the management of internal derangement of TMJ: A double-blinded randomized controlled trial	Ineligible intervention
Isik [71]	2022	https://doi.org/10.1016/j.jcms.2022.06.006	Injectable platelet-rich fibrin as treatment for temporomandibular joint osteoarthritis: A randomized controlled clinical trial	Ineligible intervention
Jacob [72]	2022	https://doi.org/10.1007/s12663-021-01519-y	Efficacy of Platelet-Rich Plasma Versus Hyaluronic Acid Following Arthrocentesis for Temporomandibular Joint Disc Disorders: A Randomized Controlled Trial	Ineligible intervention
Leketas [73]	2022	https://doi.org/10.1080/08869634.2022.2081445	Different intra-articular injection substances following temporomandibular joint arthroscopy and their effect on early postoperative period: A randomized clinical trial	Ineligible intervention
Macedo de Sousa [74]	2022	https://doi.org/10.3390/life12111739	Medium-Term Effect of Treatment with Intra-Articular Injection of Sodium Hyaluronate, Betamethasone and Platelet-Rich Plasma in Patients with Temporomandibular Arthralgia: A Retrospective Cohort Study.	Ineligible intervention
Ramakrishnan [75]	2022	https://doi.org/10.4103/njms.njms_94_20	Comparison of intraarticular injection of platelet-rich plasma following arthrocentesis, with sodium hyaluronate and conventional arthrocentesis for management of internal derangement of temporomandibular joint.	Ineligible intervention
De Sousa [76]	2020	https://doi.org/10.3390/medicina56030113	Different treatments in patients with temporomandibular joint disorders: A comparative randomized study	Ineligible intervention
Kim [77]	2020	https://doi.org/10.1186/s13063-020-04442-8	Efficacy, safety, and economic assessment of hominis placental pharmacopuncture for chronic temporomandibular disorder: A protocol for a multicentre randomized controlled trial	Ineligible publication type (study protocol)
Pihut [78]	2020	https://doi.org/10.3390/ijerph17134726	The application of intra-articular injections for management of the consequences of disc displacement without reduction	Ineligible intervention
Kutuk [79]	2019	https://doi.org/10.1097/scs.0000000000005211	Clinical and radiological comparison of effects of platelet-rich plasma, hyaluronic acid, and corticosteroid injections on temporomandibular joint osteoarthritis	Ineligible intervention
Martín-Granizo [80]	2018	https://doi.org/10.1016/j.bjoms.2018.07.004	Simple and secure intra-articular infiltration during arthroscopy of the temporomandibular joint	Ineligible intervention
Fernández-Ferro [81]	2017	https://doi.org/10.1016/j.jcms.2017.01.010	Comparison of intra-articular injection of plasma rich in growth factors versus hyaluronic acid following arthroscopy in the treatment of temporomandibular dysfunction: A randomized prospective study	Ineligible intervention
Hossameldin [82]	2017	https://doi.org/10.1016/j.ijom.2017.02.776	Efficacy of platelet-rich plasma versus hyaluronic acid intraarticular injection in arthroscopic management of Wilkes V temporomandibular joint patients	Ineligible intervention
Pihut [83]	2017	PMID: 29263455	Evaluation of remission of temporomandibular joints pain as a result of treatment of dysfunction using intra-articular injection	Ineligible intervention
Cömert Kiliç [84]	2016	https://doi.org/10.1016/j.ijom.2016.06.009	Is arthrocentesis plus platelet-rich plasma superior to arthrocentesis plus hyaluronic acid for the treatment of temporomandibular joint osteoarthritis: A randomized clinical trial	Ineligible intervention
Hegab [85]	2015	https://doi.org/10.1016/j.joms.2015.03.045	Platelet-rich plasma injection as an effective treatment for temporomandibular joint osteoarthritis	Ineligible intervention
Machoň [86]	2013	https://doi.org/10.1016/j.ijom.2013.07.694	Platelet-Rich Plasma in Temporomandibular Joint Osteoarthritis Therapy: A 3-Month Follow-Up Pilot Study	Ineligible intervention
Kargi [87]	2002	https://doi.org/10.1097/00006534-200206000-00077	Intraarticular injections of sodium hyaluronate for temporomandibular joint disorder	Ineligible intervention
